# The Remarkable Robustness of Surrogate Gradient Learning for Instilling Complex Function in Spiking Neural Networks

**DOI:** 10.1162/neco_a_01367

**Published:** 2021-03-26

**Authors:** Friedemann Zenke, Tim P. Vogels

**Affiliations:** Centre for Neural Circuits and Behaviour, University of Oxford, Oxford OX1 3SR, U.K., and Friedrich Miescher Institute for Biomedical Research, 4058 Basel, Switzerland; Centre for Neural Circuits and Behaviour, University of Oxford, Oxford OX1 3SR, U.K., and Institute for Science and Technology, 3400 Klosterneuburg, Austria

## Abstract

Brains process information in spiking neural networks. Their intricate connections shape the diverse functions these networks perform. Yet how network connectivity relates to function is poorly understood, and the functional capabilities of models of spiking networks are still rudimentary. The lack of both theoretical insight and practical algorithms to find the necessary connectivity poses a major impediment to both studying information processing in the brain and building efficient neuromorphic hardware systems. The training algorithms that solve this problem for artificial neural networks typically rely on gradient descent. But doing so in spiking networks has remained challenging due to the nondifferentiable nonlinearity of spikes. To avoid this issue, one can employ surrogate gradients to discover the required connectivity. However, the choice of a surrogate is not unique, raising the question of how its implementation influences the effectiveness of the method. Here, we use numerical simulations to systematically study how essential design parameters of surrogate gradients affect learning performance on a range of classification problems. We show that surrogate gradient learning is robust to different shapes of underlying surrogate derivatives, but the choice of the derivative’s scale can substantially affect learning performance. When we combine surrogate gradients with suitable activity regularization techniques, spiking networks perform robust information processing at the sparse activity limit. Our study provides a systematic account of the remarkable robustness of surrogate gradient learning and serves as a practical guide to model functional spiking neural networks.

## 1 Introduction

The computational power of deep neural networks (LeCun, Bengio, & Hinton, 2015; Schmidhuber, 2015) has reinvigorated interest in using in-silico systems to study information processing in the brain (Barrett, Morcos, & Macke, 2019; Richards et al., 2019). For instance, performance-optimized artificial neural networks bear striking representational similarity with the visual system (Maheswaranathan et al., 2018; McClure & Kriegeskorte, 2016; McIntosh, Maheswaranathan, Nayebi, Ganguli, & Baccus, 2016; Pospisil, Pasupathy, & Bair, 2018; Tanaka et al., 2019; Yamins & DiCarlo, 2016; Yamins et al., 2014) and serve to formulate hypotheses about their mechanistic underpinnings. Similarly, the activity of artificial recurrent neural networks optimized to solve cognitive tasks resembles cortical activity in prefrontal (Cueva et al., 2019; Mante, Sussillo, Shenoy, & Newsome, 2013), medial frontal (Wang, Narain, Hosseini, & Jazayeri, 2018), and motor areas (Michaels, Schaffelhofer, Agudelo-Toro, & Scherberger, 2019; Stroud, Porter, Hennequin, & Vogels, 2018), thus providing us with new vistas for understanding the dynamic properties of computation in recurrent neural networks (Barrett et al., 2019; Sussillo & Barak, 2012; Williamson, Doiron, Smith, & Yu, 2019).

All of these studies rely on conventional artificial neural networks with graded activation functions as commonly used in machine learning. The recipe for building a deep neural network is straightforward. The value of a scalar loss function defined at the output of the network is decreased through gradient descent. Deep neural networks differ from biological neural networks in important respects. For instance, they lack cell type diversity and do not obey Dale’s law while ignoring the fact that the brain uses spiking neurons. We generally accept these flaws because we do not know how to construct more complicated networks. For instance, gradient descent works only when the involved system is differentiable. This is not the case for spiking neural networks (SNNs).

Surrogate gradients have emerged as a solution to build functional SNNs capable of solving complex information processing problems (Bellec, Salaj, Subramoney, Legenstein, & Maass, 2018; Cramer, Stradmann, et al., 2020; Esser et al., 2016; Hunsberger & Eliasmith, 2015; Lee, Delbruck, & Pfeiffer, 2016; Neftci, Mostafa, & Zenke, 2019; Pfeiffer & Pfeil, 2018; Shrestha & Orchard, 2018). To that end, the actual derivative of a spike, which appears in the analytic expressions of the gradients, is replaced by any well-behaved function. There are many possible choices of such surrogate derivatives, and consequently, the resulting surrogate gradient is, unlike the true gradient of a system, not unique. A number of studies have successfully applied different instances of surrogate derivatives to various problem sets (Bellec et al., 2018; Esser et al., 2016; Huh & Sejnowski, 2018; Shrestha & Orchard, 2018; Woźniak, Pantazi, Bohnstingl, & Eleftheriou, 2020; Zenke & Ganguli, 2018). While this suggests that the method does not crucially depend on the specific choice of surrogate derivative, we know relatively little about how the choice of surrogate gradient affects the effectiveness and whether some choices are better than others. Previous studies did not address this question because they solved different computational problems, thus precluding a direct comparison. In this letter, we address this issue by providing benchmarks for comparing the trainability of SNNs on a range of supervised learning tasks and systematically vary the shape and scale of the surrogate derivative used for training networks on the same task.

## 2 Results

To systematically evaluate the performance of surrogate gradients, we sought to repeatedly train the same network on the same problem while changing the surrogate gradient. Toward this end, we required a demanding spike-based classification problem with a small computational foot-print to serve as benchmark. There are only few established benchmarks for SNNs. One approach is to use analog-valued machine learning data sets as input currents directly (Hunsberger & Eliasmith, 2015) or to first convert them to Poisson input spike trains (Lee et al., 2016; Pfeiffer & Pfeil, 2018). These input paradigms, however, do not fully capitalize on the ability to encode information in spike timing, an important aspect of spiking processing. Gütig (2016) addressed this point with the Tempotron by classifying randomly generated spike timing patterns in which each input neuron fires a single spike. Yet completely random timing precludes the possibility of assessing generalization performance, that is, the ability to generalize to previously unseen inputs.

### 2.1 Random Manifolds as Basis for Flexible Benchmarks

To assess if SNNs could learn to categorize spike patterns and generalize to unseed patterns, we created a number of synthetic classification data sets with added temporal structure. Specifically, we created spike rasters for a given set of input afferents. Each afferent only fired one spike, and the spike times of all afferents were constrained to lie on a low-dimensional smooth, random manifold in the space of all possible spike timings. All data points from the same manifold were defined as part of the same input class, whereas different manifolds correspond to different classes.

**Figure 1:**
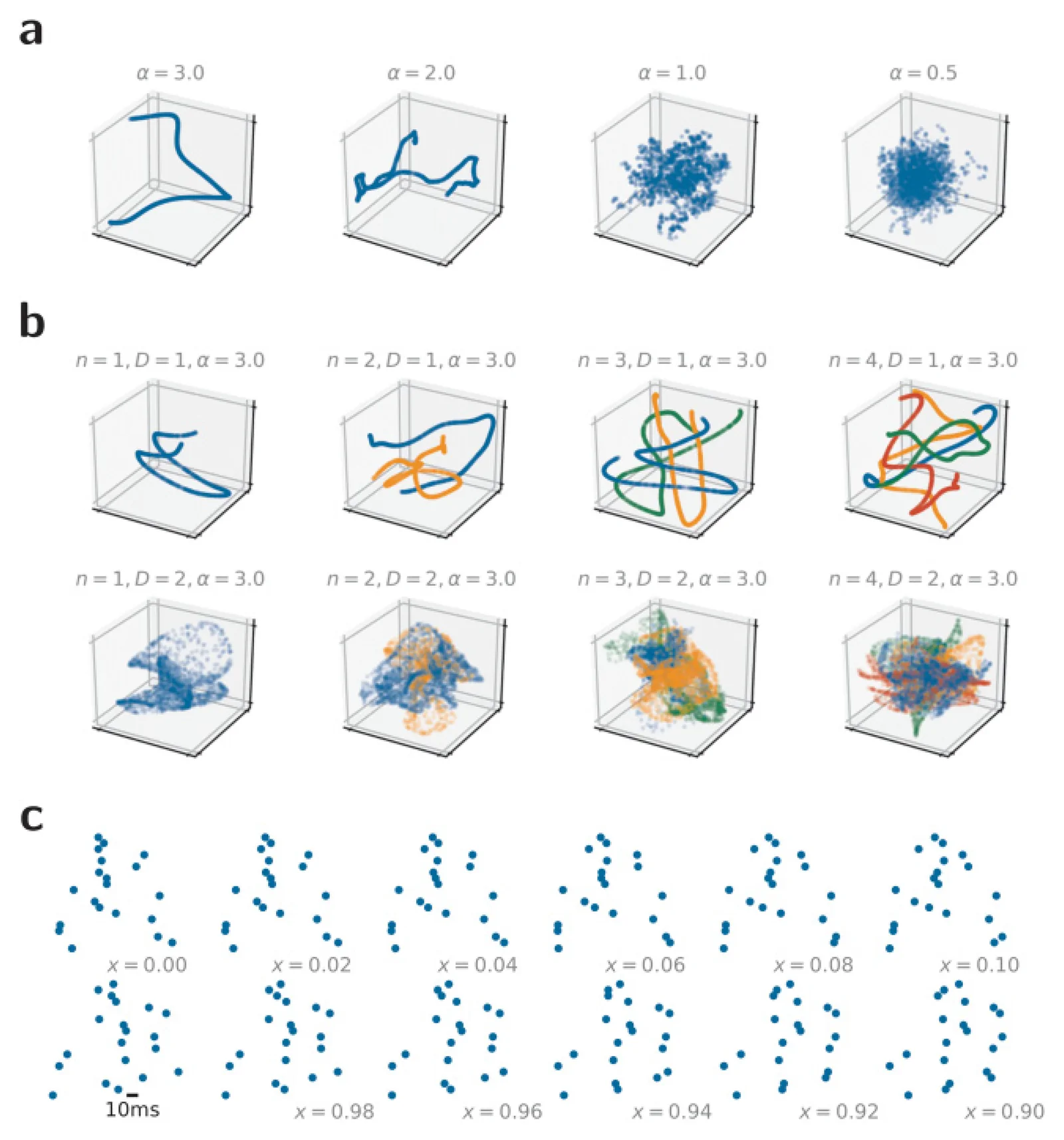
Smooth random manifolds provide a flexible way of generating synthetic spike-timing data sets. (a) Four one-dimensional example manifolds for different smoothness parameters *α* in a three-dimensional embedding space. From each manifold, we plotted 1000 random data points. (b) Same as in panel a, but keeping *α* = 3 fixed while changing the manifold-dimension *D* and the number of random manifolds (different colors). By sampling different random manifolds, it is straight-forward to build synthetic multiway classification tasks. (c) Spike raster plots corresponding to 12 samples along the intrinsic manifold coordinate *x* of a one-dimensional smooth random manifold (*α*=3) whereby we interpreted the embedding space coordinates as firing times of the individual neurons.

The spike-timing manifold approach has several advantages: First, the temporal structure in the data permits studying generalization, a decisive advantage over using purely random spike patterns. Second, the task complexity is seamlessly adjustable by tuning the number of afferents, manifold’s smoothness parameter *α* (see Figure 1a), the intrinsic manifold dimension *D*, and the number of classes *n* (see Figure 1b). Third, we ensure that each input neuron spikes exactly once (see Figure 1c), guaranteeing that the resulting data sets are purely spike-timing-dependent and thus cannot be classified from firing rate information. Finally, sampling an arbitrary number of data points from each class is computationally cheap, and it is equally easy to generate an arbitrary number of different data sets with comparable properties.

**Figure 2:**
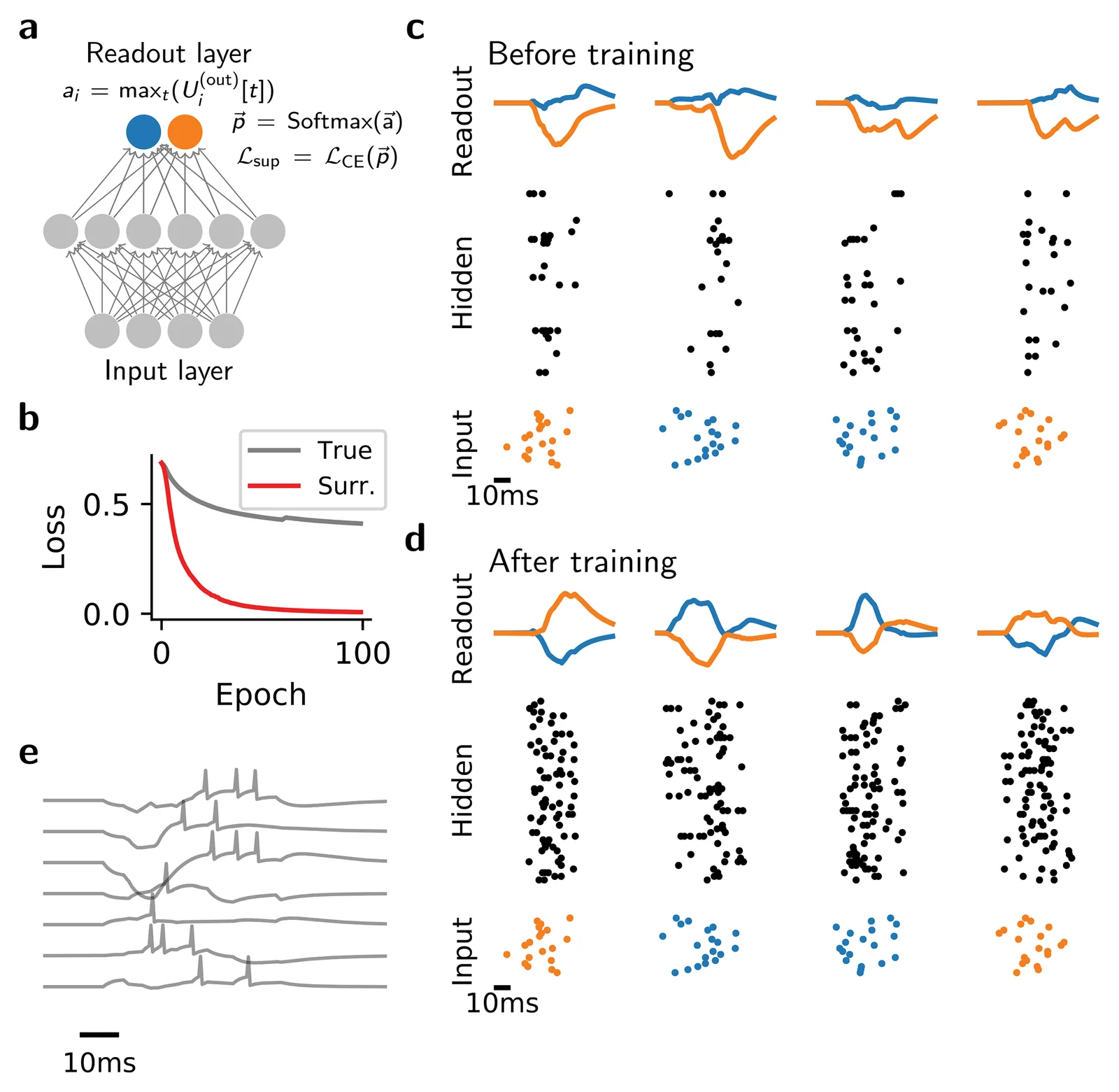
Surrogate gradient descent allows building functional SNNs. (a) Sketch of the network model with two readout units at the top. The supervised loss function *ℒ*_sup_ is defined by first taking the maximum activation over time of the readout units ${𝒰}_{i}^{\text{out}}$ (orange and blue) and then applying a Soft-max and cross-entropy loss *ℒ*_CE_ (see section 4 for details). (b) Learning curves of the network when using the actual gradient (“true,” gray) or a surrogate gradient (red) to train an SNN on a binary random manifold classification problem. (c) Snapshot of network activity before training. Bottom: Spike raster of the input layer activity. Four different inputs corresponding to two different classes are plotted in time (orange/blue). Middle: Spike raster of the hidden-layer activity. Top: Readout unit membrane potential. The network erroneously classifies the two “orange” inputs as belonging to the “blue” class, as can be read off from the maximum activity of its readout units. (d) Same as in panel c, but following training of the network with surrogate gradient descent. (e) Example membrane potential traces from seven randomly selected hidden-layer neurons during a single trial.

To demonstrate the validity of our approach, we tested it on an SNN with a single hidden layer on a simple two-way classification problem (see Figure 2a and section 4). We modeled the units of the hidden layer as current-based leaky integrate-and-fire neurons. Between layers, the connectivity was strictly feedforward and all-to-all. The output layer consisted of two leaky integrators that did not spike, allowing us to compute the maximum of the membrane potential (Gütig & Sompolinsky, 2006) and interpret these values as the inputs for a standard classification loss function for supervised learning (see section 4). In this setup, the readout unit with the highest activity level signals the putative class-membership of each input (Cramer, Stradmann, Schemmel, & Zenke, 2020).

We first confirmed that learning is poor when we used the actual gradient. To that end, we computed it using the derivative of the hard threshold nonlinearity of the spikes. As expected, the hard threshold nonlinearity prevented gradient flow into the hidden layer (Neftci et al., 2019) and consequently led to poor performance (see Figures 2b and 2c). In contrast, when we used surrogate gradients to train the same network, the problem disappeared. Learning took place in both the hidden and output layers and resulted in a substantial reduction of the loss function (see Figures 2b to 2e).

### 2.2 Surrogate Gradient Learning Is Robust to the Shape of the Surrogate Derivative

**Figure 3:**
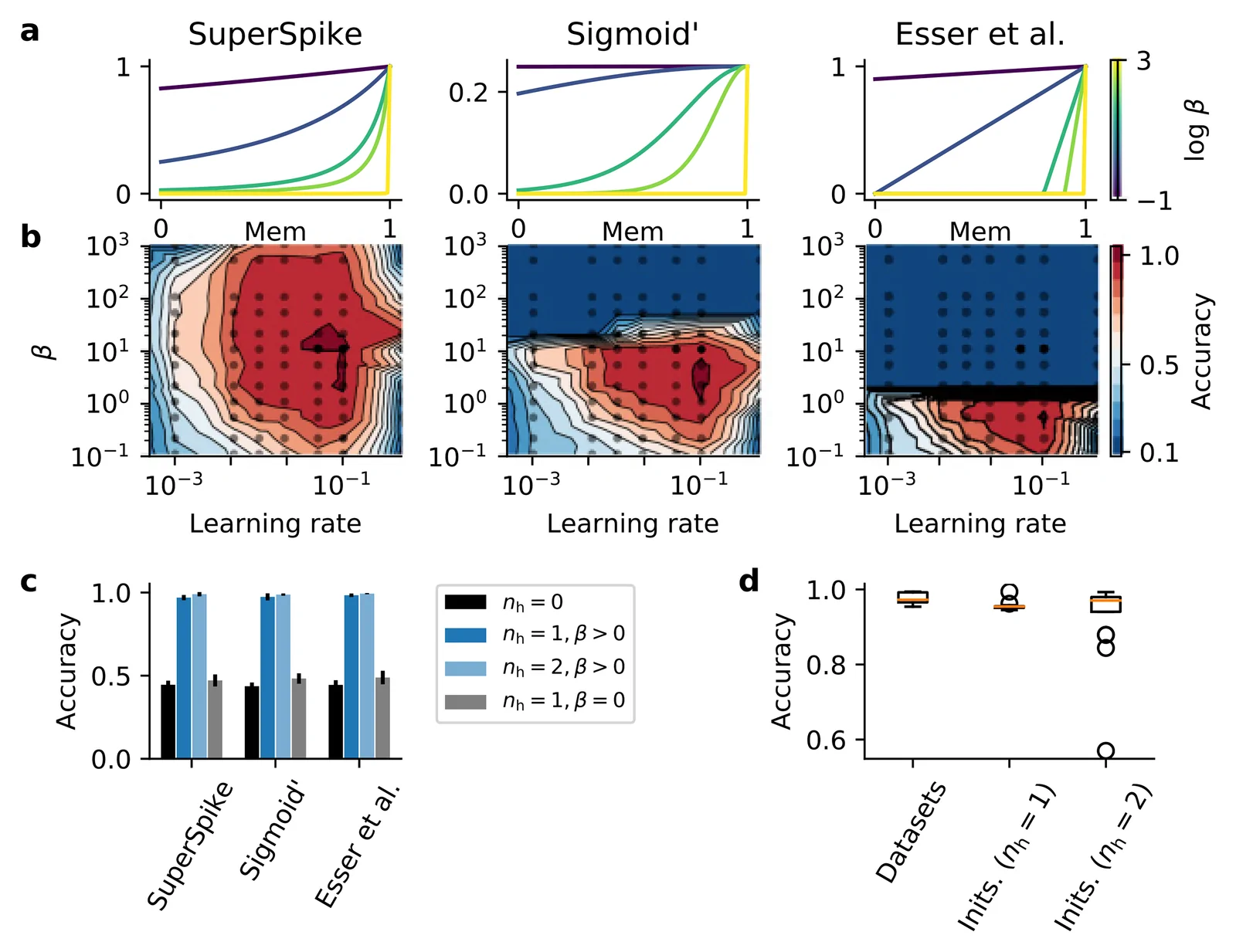
Surrogate gradient learning is robust to the shape of the surrogate derivative. (a) Three different surrogate derivative shapes that have been used for training on the synthetic smooth random manifold spiking data set. From left to right: SuperSpike (Zenke & Ganguli, 2018), the derivative of a fast sigmoid function, Sigma′, the derivative of a standard sigmoid function, and “Esser et al.,” a piece-wise linear function (Bellec et al., 2018; Esser et al., 2016). Colors correspond to different values of the slope parameter *β*. (b) Accuracy on held-out data as a function of the learning rate *η* and the slope *β* for the corresponding surrogates in panel a for a network with one hidden layer (*n*_*h*_ = 1). (c) Test accuracy for the five best parameter combinations obtained from a grid search, as shown in panel b for different surrogates and numbers of hidden layers *n*_*h*_. While a network without a hidden layer is unable to solve the classification problem (black), networks trained with a wide range of different surrogates and slope parameters (*β* > 0) have no problem solving the task with high accuracy (shades of blue). However, the problem is not solved with high accuracy by a network with a hidden layer in which the surrogate derivative was a constant during training (*β* = 0; gray). Error bars correspond to the standard deviation (*n* = 5). (d) Whisker plot of classification accuracy for a network with one hidden layer over five different realizations of the random manifold data sets (“Datasets”) and for the same data set, but using different weight initializations (“Inits.”) in networks with either one (*n*_h_ = 1) or two (*n*_h_ = 2) hidden layers.

A necessary ingredient of surrogate gradient learning is a suitable surrogate derivative. To study the effect of the surrogate derivative comparably, we generated a random manifold data set with 10 classes. We chose the remaining parameters, that is, the number of input units, the manifold dimension, and the smoothness *α* to make the problem impossible to solve for a network without a hidden layer while at the same time keeping the computational burden minimal. We trained multiple instances of the same network with a single hidden layer (*n*_h_ = 1) using the derivative of a fast sigmoid as a surrogate derivative (see Figure 3a “SuperSpike”; Zenke & Ganguli, 2018) on this data set. In each run, we kept both the data set and the initial parameters of the model fixed but varied the slope parameter *β*, of the surrogate. For each value of *β*, we performed a parameter sweep over the learning rate *η*. Following training, we measured the classification accuracy on held-out data. This search revealed an extensive parameter regime of *β* and *η* in which the system was able to solve the problem with high accuracy (see Figure 3b). The addition of a second hidden layer only marginally improved on this result, and, as expected, a network without a hidden layer performed poorly (see Figure 3c). The extent of the parameter regime yielding high performance suggests remarkable robustness of surrogate gradient learning to changes in the steepness of the surrogate derivative. While a steep approach to the threshold could be seen as a closer, hence better, approximation of the actual derivative of the spike, the surrogate gradient remains largely unaffected by how closely the function resembles the exact derivative as long as it is not a constant.

Next we tested different surrogate derivative shapes, namely a standard sigmoid (Sigmoid′) and piece-wise linear function (Esser et al., 2016; see Figure 3a; Bellec et al., 2018). This manipulation led to a reduction of the size of the parameter regime in *β* in which the network was able to perform the task, which is presumably due to vanishing gradients (Hochreiter, 1998). However, there was no substantial reduction in maximum performance (see Figures 3b and 3c). Using a piece-wise linear surrogate derivative (Esser et al.) led to a further reduction of viable parameters *β* (see Figure 3b), but did not affect maximum performance, regardless of whether we used one or two hidden layers (see Figure 3c). To check whether a surrogate derivative was required at all for solving the random manifold problem, we assayed the learning performance for *β* = 0, which corresponds to setting the function to 1. This change resulted in a significant drop in performance comparable to a network without hidden units (see Figure 3c) suggesting that a nonlinear voltage dependence is crucial to learn useful hidden-layer representations. Finally, we confirmed that these findings were robust to different initial network parameters and data sets (see Figure 3d) apart from only a few outliers with low performance in the two-hidden-layer case (*n*_*h*_ = 2). These outliers point at the vital role of proper initialization (He, Zhang, Ren, & Sun, 2015; Mishkin & Matas, 2016).

### 2.3 Surrogate Gradient Learning Is Sensitive to the Scale of the Surrogate Derivative

**Figure 4:**
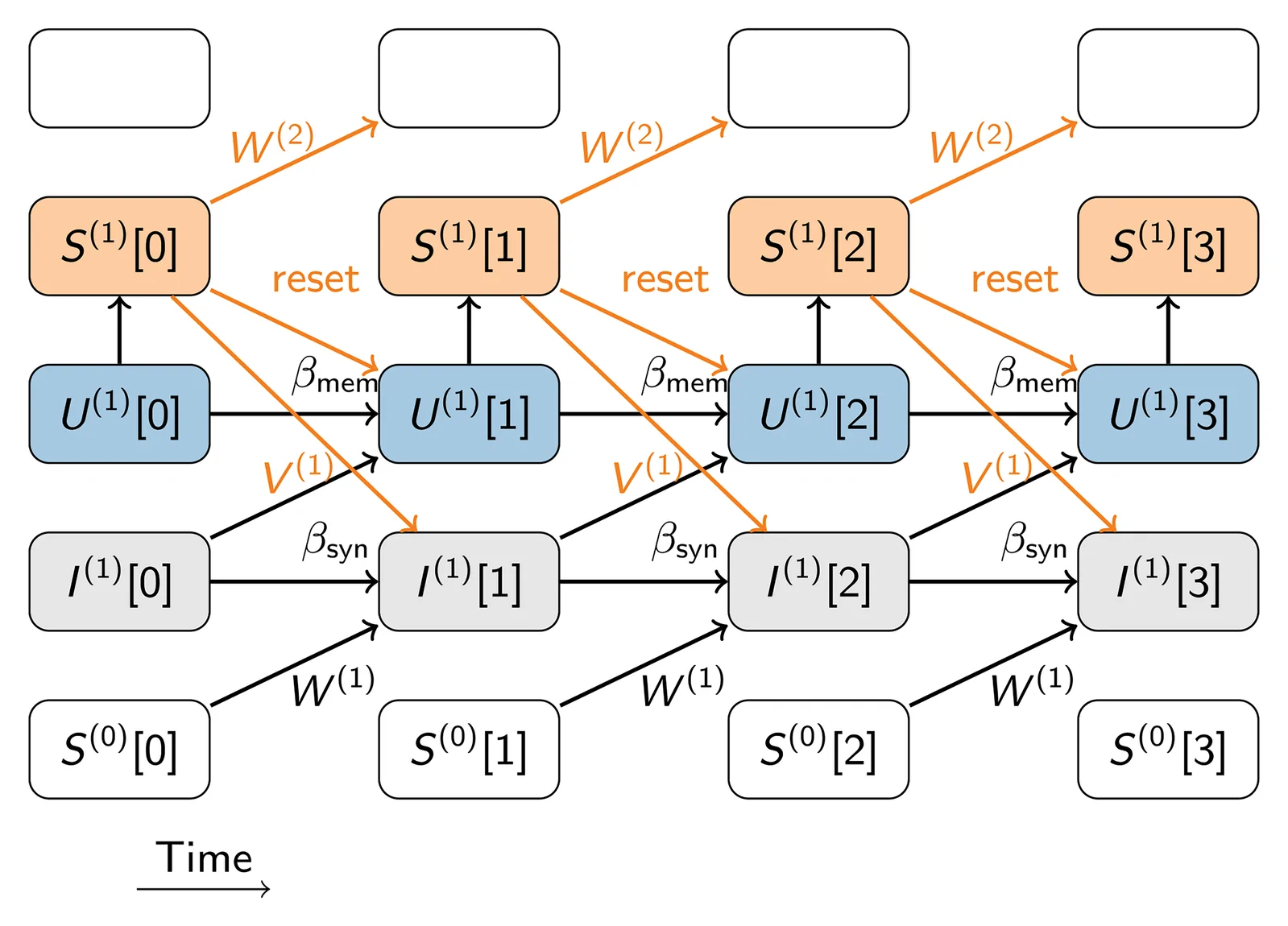
SNNs can have both implicit and explicit recurrence. Schematic of the computational graph of a single SNN layer composed of leaky integrate-and-fire (LIF) neurons (see section 4). Input spike trains *S*^(0)^ enter at the bottom and affect the synaptic current variable *I*^(1)^ through the feedforward weights *W* ^(1)^. Time flows from left to right. Any link that connects temporally adjacent nodes in the graph constitutes a form of recurrence in the computation whereby the synaptic connections *V* ^(1)^ contribute explicit recurrence to the graph. Implicit recurrence is contributed, for instance, by the decay of synaptic current variables and the membrane potentials *U* ^(1)^. Additionally, the spike reset contributes another form of implicit recurrence by coupling the future states to the output spike train *S*^(1)^. Recurrences involving the surrogate derivative (e.g. the reset) depend on both the shape and the scale of the surrogate chosen and can substantially alter the surrogate gradient.

**Figure 5:**
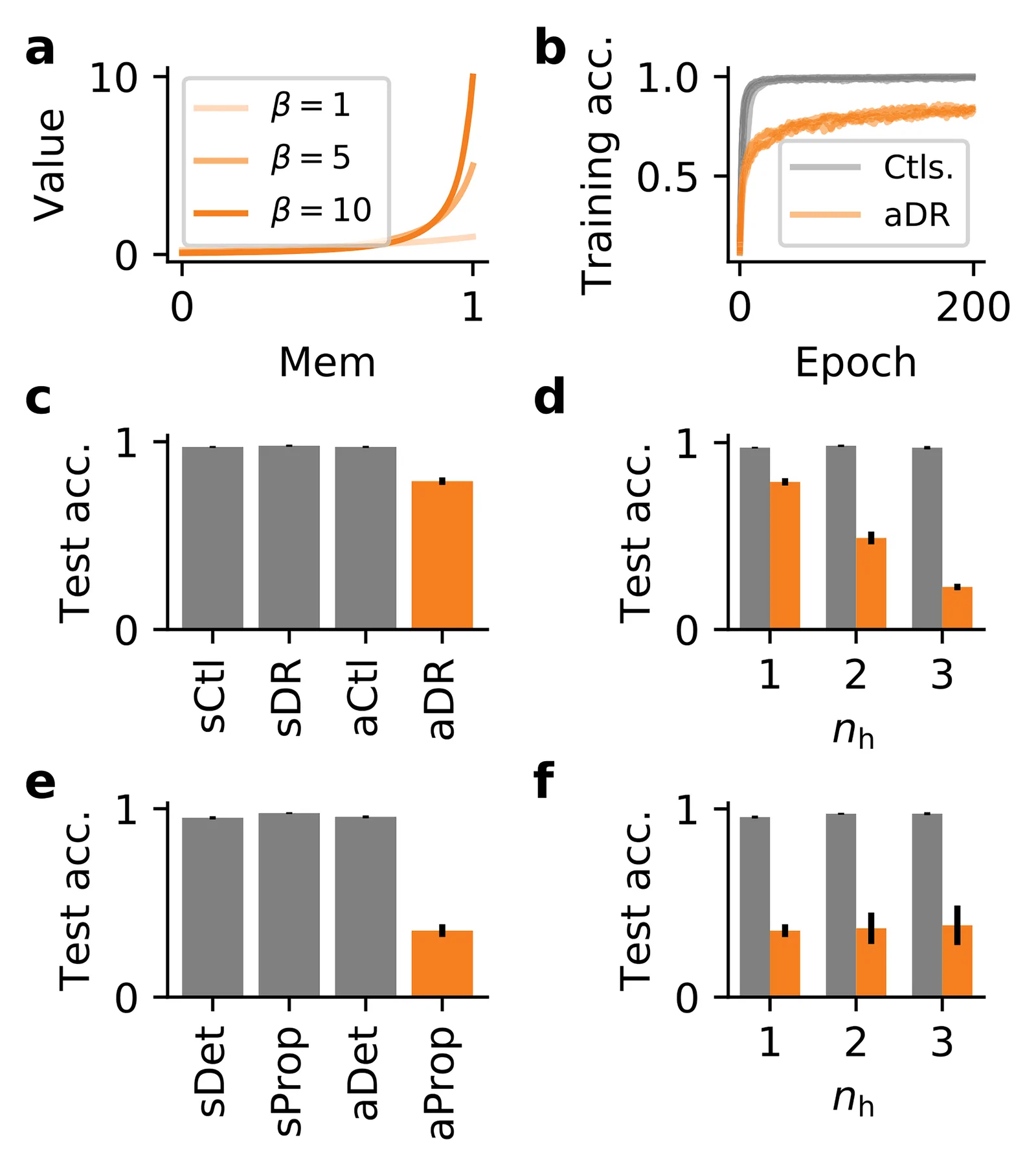
Surrogate gradient learning is sensitive to the scale of the surrogate derivative. (a) Illustration of pseudo-derivatives *σ*′ that converge toward the actual derivative of a hard spike threshold *β* → ∞. Note that in contrast to Figure 3a, their maximum value grows as *β* increases. (b) Training accuracy of several spiking networks (*n*_*h*_ = 1) during training on a synthetic classification task. The gray curves comprise control networks in which the surrogate derivative was either normalized to one or in which we used an asymptotic surrogate derivative but prevented surrogate gradients from flowing through the spike reset. Orange curves correspond to networks with asymptotic pseudo-derivatives with differentiable spike reset (aDR). In all cases, we plot the five best-performing learning curves obtained from an extensive grid search over *β* and the learning rate *η* (cf. Figure 3). (c) Quantification of the test accuracy of the different learning curves shown in panel b. We trained all networks using a SuperSpike nonlinearity. The reset term was either ignored (sCtl) or a differentiable reset was used (aDR). Similarly, we considered an asymptotic variant of SuperSpike that does converge toward the exact derivative of a step function for *β* → ∞, without (aCtl) or with a differentiable reset term (aDR). The results shown correspond to the 10 best results from a grid search. The error bars denote the standard deviation. (d) A similar comparison of control cases in which reset terms were ignored (gray) or could contribute to the surrogate gradient (orange) for different numbers of hidden layers. (e) Test accuracy as in panel c, but comparing SuperSpike s and the asymptotic a case in which gradients can flow through recurrent connections (Prop) versus the detached case (Ctl). (f) Test accuracy for asymptotic SuperSpike as a function of the number of hidden layers for networks in which gradients were flowing through recurrent connections (orange) versus the detached case (gray).

In most studies that rely on surrogate gradients, the surrogate derivative is normalized to 1 (Bellec et al., 2018; Esser et al., 2016; Neftci et al., 2019; Shrestha & Orchard, 2018; Zenke & Ganguli, 2018) (see Figure 3a), markedly different from the actual derivative of a spiking threshold which is infinite (see Figure 5a). The scale of the substituted derivative has a strong effect on the surrogate gradient due to both explicit and implicit forms of recurrence in SNNs (see Figure 4; Neftci et al., 2019). Most notably, the scale may determine whether gradients vanish or explode (Hochreiter, 1998). However, it is unclear to what extent such differences translate into performance changes of the trained networks.

To gain a better understanding of how derivative scales larger than one affect surrogate gradient learning, we trained networks on a fixed random manifold task (see Figures 3a to 3c), using an asymptotic version of the SuperSpike surrogate (aCtl; see Figure 3) and our well-tested standard Super-Spike function (sCtl; see Figure 3) as a control (sCtl). As we expected, the difference in scale to manifest itself primarily in the presence of recurrence, we compared networks in which we treated the spike reset as differentiable (DR) with networks in which its contribution was ignored by detaching it from the computational graph. Technically speaking, we prevented PyTorch’s auto-differentiation routines (Paszke et al., 2019) from considering connections in the computational graph that correspond to the spike reset when computing the gradient with backpropagation through time (BPTT) (see equation 4.1). While both the normalized (sCtl) and the asymptotic surrogate (aCtl) performed equally well when the reset was detached, combining a differentiable reset with an asymptotic surrogate derivative (aDR) led to impaired performance (see Figures 5b and 5c). This adverse effect on learning was amplified in deeper networks (see Figure 5d). Thus, the scale of the surrogate derivative plays an important role in learning success if implicit recurrence, as contributed by the spike reset, is present in the network.

Since the spike reset constitutes a specific form of implicit recurrence (cf. see Figure 4), we were wondering whether we would observe a similar phenomenon for explicit recurrence through recurrent synaptic connections. To that end, we repeated the performance measurements in networks with recurrent connections but kept the spike reset term detached to prevent gradient flow. We observed a small but measurable reduction in accuracy for the best-performing networks (see Figures 5c and 5e). Importantly, however, there was a substantial decrease in classification performance when gradients were allowed to flow through recurrent connections and the asymptotic SuperSpike variant was used (see Figure 5e). Unlike in the differentiable reset (DR) case, the effect was severe enough to drop network performance to chance level even for a network with a single hidden layer (see Figure 5f). In summary, surrogate gradients are sensitive to the scale of the surrogate derivative. More specifically, when the scale of the surrogate derivative is too large, and either implicit or explicit recurrence is present in the network, the effect on learning can be detrimental.

### 2.4 Surrogate Gradient Learning Is Robust to Changes in the Loss Functions, Input Paradigms, and Data Sets

**Figure 6:**
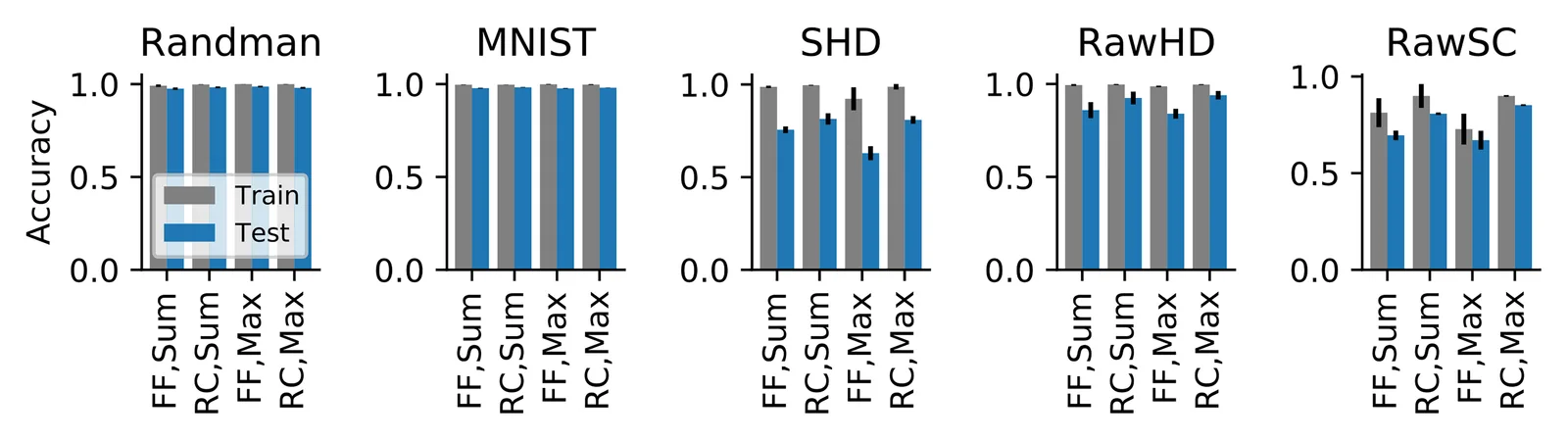
Surrogate gradient learning is effective on different loss functions, input paradigms, and data sets. Bar plots showing the test classification accuracy for different data sets (a–e). The plots further distinguish models by their readout configuration (Sum versus Max) and whether they use purely feedforward (FF) or explicitly recurrent (RC) synaptic connectivity. Each bar corresponds to the mean over the 10 best-performing models on held-out validation data, and error bars signify the standard deviation.

So far we have investigated synthetic random manifold data sets in strictly feedforward networks trained with loss functions that were defined on the maximum over time (Max) of the readout units. Next, we performed additional simulations in which the loss was computed by summation over time (“Sum”; see section 4). Based on our findings above, we limited our analysis to the SuperSpike surrogate with *β* = 10 and detached reset terms. As before, we performed a grid search over the learning rate *η* and selected the 10 best-performing models using held-out validation data. We then computed their classification performance on a separate test set. We repeated our simulation experiments on the above random manifold task and did not observe any substantial difference in the accuracy for the Max and Sum type readout heads (see Figure 6a).

**Figure 7:**
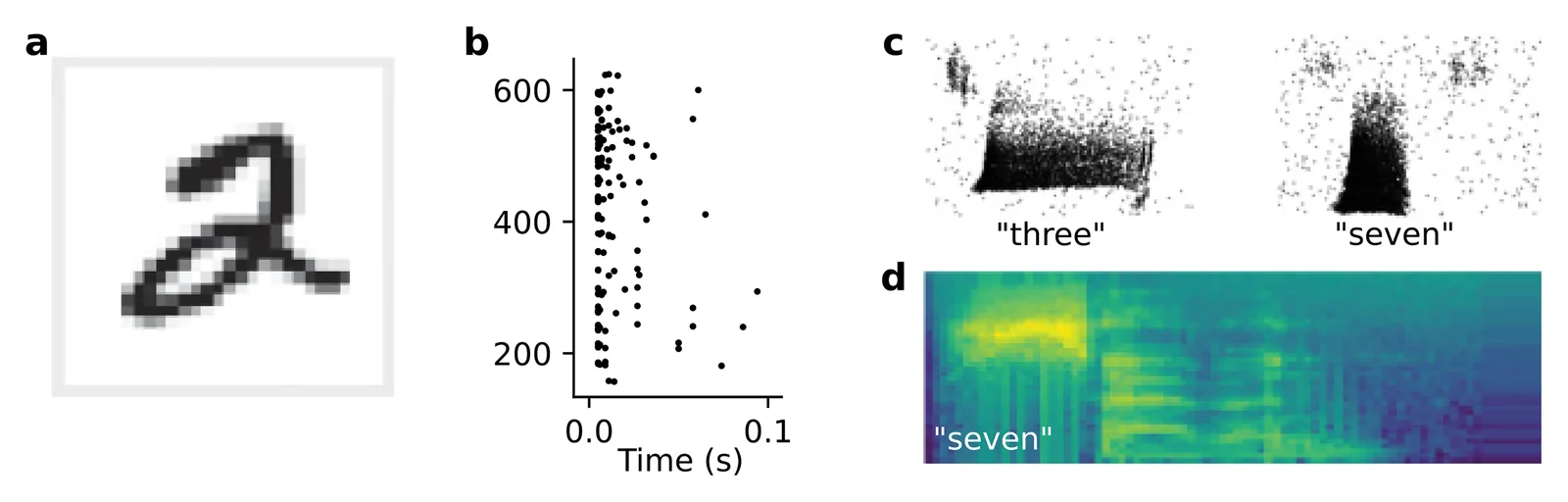
Examples of different input paradigms. (a) One example image from MNIST handwritten digit data set. (b) Spike raster plot of the corresponding spike latency encoding for the 28 × 28 = 784 input neurons. (c) Spike raster of two example inputs for the spoken digits “three” and “seven” taken from the SHD data set (Cramer, Stradmann et al., 2020). (d) Mel-scaled spectrogram of an utterance of the number “seven” as used for simulations using raw audio input.

To check the validity of these findings on a different data set, we trained networks on MNIST by converting pixel values into spike latencies (see Figures 7a and 7b and section 4). In this paradigm, each input neuron fires either a single or no spike for each input. The networks reached a test accuracy of (98.3 ± 0.9) %, which is comparable to a conventional artificial neural network with the same number of neurons and hidden layers and to previous SNN studies using temporal coding (Mostafa, 2018). We did not observe any discernible performance differences between the two readout types we tested (see Figure 6b).

Further, to study the effect of explicit recurrence, we ran separate experiments for recurrently connected networks (RC). Importantly, we did not observe any substantial performance differences between strictly feedforward and recurrently connected networks either (see Figures 6a and 6b).

We speculated that the absence of an effect may be due to the short duration of the input spike trains considered so far (approximately 50 ms). Recurrent connections are typically thought to provide neural networks with longer timescale dynamics, effectively giving the network a working memory. Thus, the beneficial effects of recurrent connections may only emerge when using stimuli of longer duration, with potentially multiple spikes from any given input neuron.

To test this hypothesis, we trained networks on the Spiking Heidelberg Digits (SHD) data set (Cramer, Stradmann et al., 2020), which consists of simulated input spikes from the auditory pathway of varied duration between 0.6 and 1.4 s (see Figure 7c). Indeed, we found that the best-performing models in this case were recurrent and achieving state-of-the-art classification accuracy of (0.82 ± 0.02) % (see Figure 6c). These data are consistent with the notion that working memory plays a vital role in classifying longer input patterns.

### 2.5 Surrogate Gradient Learning in Networks with Current-Based Input

Until now, we have considered spike-based data sets. While spiking inputs are arguably the most natural input to SNNs, they come with an important caveat. All spiking data sets assume a specific encoding model, which is used to convert analog input data into a spiking representation. The chosen model, however, may not be optimal and thus adversely affect classification performance. To avoid this issue, we sought to learn the spike encoding by directly feeding current-based input to a set of spiking units (Zimmer, Pellegrini, Singh, & Masquelier, 2019). To test this idea, we converted the raw audio data of the Heidelberg Digits (Cramer, Stradmann et al., 2020) to Mel-spaced spectrograms (see Figure 7d and section 4). To reduce overfitting, we decreased the number of channels and time steps to values commonly used in artificial speech recognition systems. Specifically, we used 40 channels and 80 time frames, corresponding to compression in time by a factor of about five (see section 4). The networks trained on this RawHD data set showed reduced overfitting yet still benefited from recurrent connections compared to strictly feedforward networks (see Figure 6d). Concretely, recurrent networks reached (94 ± 2) % test accuracy, whereas the feedforward networks reached only (85 ± 3) %. In agreement with the results on Randman and MNIST, there were no significant differences between the Sum and Max readout configurations (see Figure 6).

We wanted to know whether the discrepancy between feedforward and recurrent networks would increase for more challenging data sets. This was made possible by the performance gain from reducing the input dimension to 40 channels, which allowed us to train SNNs on the larger Speech Command data set (Warden, 2018; see section 4). This data set contains over 100,000 utterances from 35 classes, including “yes,” “no,” and “left.” In contrast to the original intended use for keyword spotting, here we assayed its top-1 classification accuracy over all classes, a more challenging problem than accurate detection of only a subset of words. The best SNNs achieved (85.3 ± 0.3) % on this challenging benchmark (see Figure 6e). On the same task, the spiking feedforward network performed at (70 ± 2) %. There was a clear benefit from adding recurrent connections to the network, with the performance of the Max ((85.3 ± 0.3) %) being slightly better than the Sum ((80.7 ± 0.4) %) readout configuration.

These findings illustrate that surrogate gradient learning is robust to changes in the input paradigm, including spiking and nonspiking data sets. For more complex data sets, recurrently connected networks performed better than strictly feedforward networks. Finally, in the majority of cases, surrogate gradient learning was robust to the details of how the output loss was defined.

### 2.6 Optimal Sparse Spiking Activity Levels in SNNs

**Figure 8:**
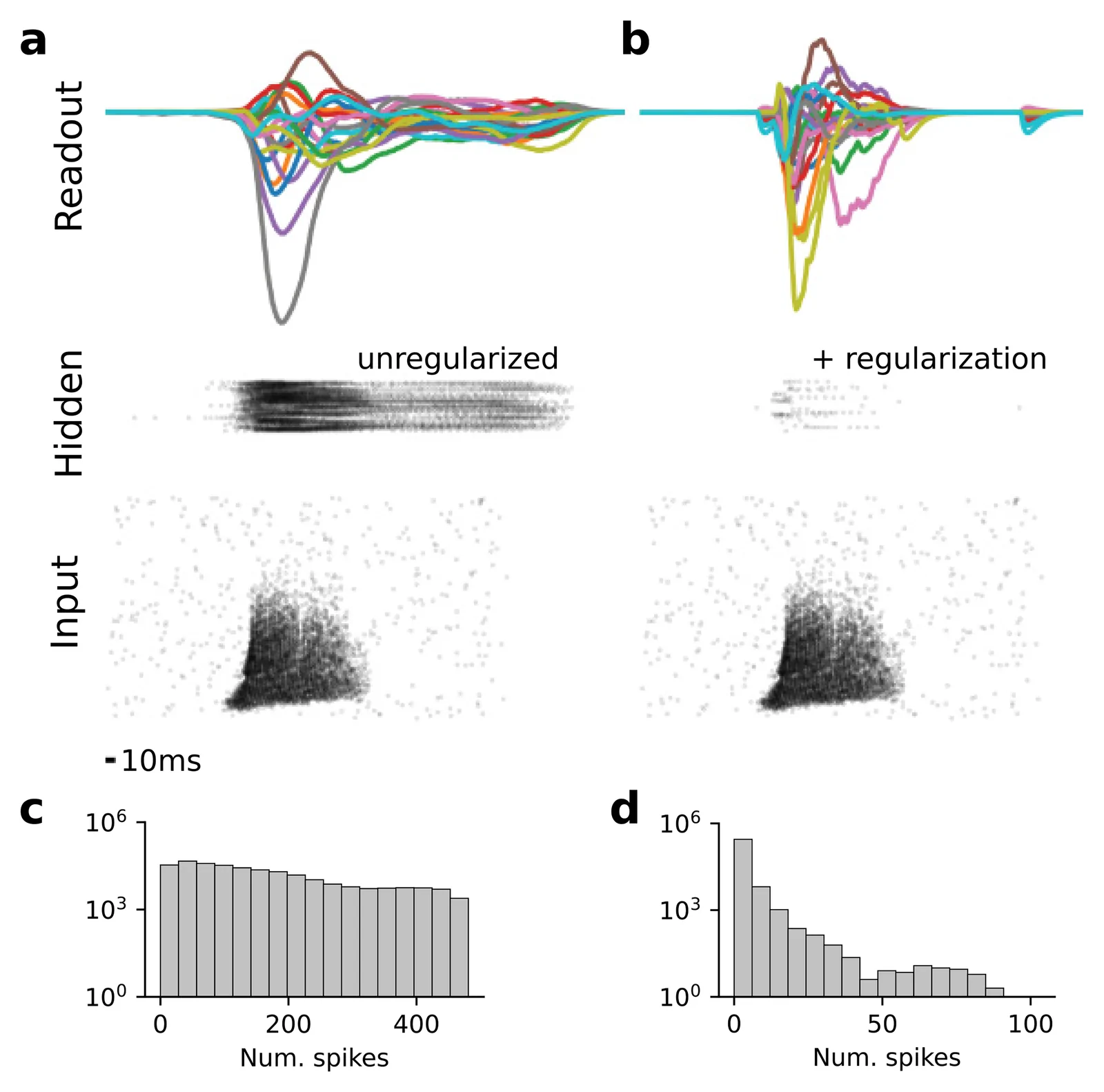
Activity regularization renders hidden-layer activity sparse while maintaining functionality. (a) Activity snapshot of one example input from SHD in a trained network. Spike raster plots of the input and hidden-layer units are shown at the bottom and in the middle. The activity of the 20 readout units is plotted at the top, with the brown line corresponding to the correct output for this example. Without any specific regularization, spiking activity in the hidden layer is pathologically high. (b) As in panel (a), but for a network trained with a penalty term for high spiking activity. This form of activity regularization drastically alters the hidden-layer activity for the same input, while leaving the winning output of the network unchanged (brown line). (c) Distribution of the number of spikes emitted by individual hidden neurons in the unregularized network over all trials of the test data set. The maximum firing rate in this simulation was 500 Hz. (d) Same as in panel c, but for the regularized network.

Up to now, we have focused on maximizing classification accuracy while ignoring the emerging activity levels in the resulting SNNs. However, we found that for some solutions, the neurons in these networks displayed implausibly high firing rates (see Figure 8a). Experimental results suggest that most biological networks exhibit sparse spiking activity, a feature that is presumed to underlie their superior energy efficiency (Boahen, 2017; Cramer, Billaudelle et al., 2020; Neftci, 2018; Roy, Jaiswal, & Panda, 2019; Schemmel et al., 2010; Sterling & Laughlin, 2017).

We investigated whether surrogate gradients could instantiate SNNs in this biologically plausible, sparse activity regime. To that end, we trained SNNs with added activity regularization that penalized high spiking activity (see Figure 8b and section 4) and recorded their hidden-layer activity. While neurons in the unregularized network frequently emityed the maximally possible number of 500 spikes per trial (see Figure 8c), regularization drastically reduced both the maximal spike count per event and the overall probability of high spike count events (see Figure 8d).

**Figure 9:**
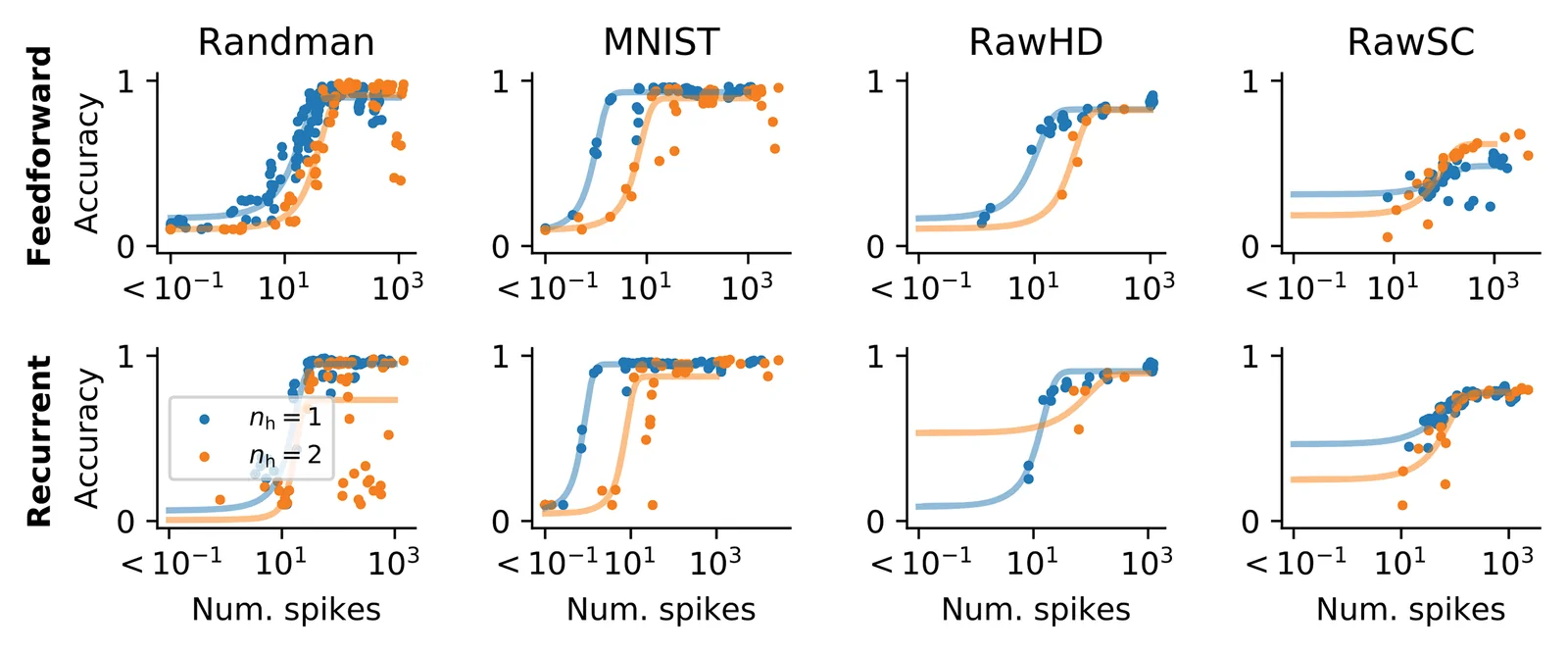
Classification accuracy degrades below a critical number of hidden-layer spikes. Plots showing classification accuracy as a function of the average number of hidden-layer spikes per input. The different columns correspond to the different data sets (see Figures 6 and 7). Top row: Networks with feedforward connectivity. Bottom row: Networks with recurrent synapses. Blue data points correspond to networks with one hidden layer, whereas orange data points come from networks with two hidden layers. The solid lines correspond to fitted sigmoid functions.

Despite a sizable reduction in spike count, many networks retained high classification accuracy. In most cases, the number of spikes could be reduced by approximately two orders of magnitude before there was a notable decline in performance, and we found a critical transition in the average number of hidden-layer spikes below which networks performed poorly (see Figure 9). For example, in the random manifold task, the transition occurred at approximately 36 hidden-layer spikes per input in a single hidden layer and approximately 76 spikes in a two-hidden-layer feed-forward network. In the recurrent network, this number was reduced to 26 (*n*_h_ = 1).

In the case of MNIST, fewer than 10 spikes were sufficient on average to achieve the point of diminishing returns beyond which additional spiking activity did not improve classification performance.

This trend could be replicated for all other data sets, with varying degrees of spike reduction. Adding a hidden layer generally required more spikes for the same performance. Recurrency generally did not have a great effect on the minimum number of spikes and did not improve performance except on RawSC. On RawSC, 80% classification accuracy was achieved only by a few feedforward networks with more than 2000 spikes on average. In the recurrent network, this level was already attained with about 150 spikes (see Figure 9).

In all cases, the transition from chance level to maximum accuracy occurred in less than one order of magnitude change in the mean number of spikes. On all data sets we tested, the addition of a second hidden layer led to an overall increase in mean spiking activity, which did not yield a notable performance change on Randman and MNIST but resulted in small improvements on RawHD and RawSC.

These results illustrate that activity-regularized SNNs can perform with high accuracy down to some critical activity threshold at which their performance degrades rapidly. Importantly, we found several network configurations that showed competitive performance with an average number of spikes substantially lower than the number of hidden units. For instance, to classify MNIST with high accuracy, an average of 10 to 20 action potentials was sufficient. Such low activity levels are more consistent with the sparse neuronal activity observed in biological neural circuits and illustrate that surrogate gradients are well suited to building SNNs that use such plausible sparse activity levels for information processing.

## 3 Discussion

Surrogate gradients offer a promising way to instill complex functions in artificial models of spiking networks. This step is imperative for developing brain-inspired neuromorphic hardware and using SNNs as in silico models to study information processing in the brain. In this letter, we have focused on two aspects of surrogate gradient learning in SNNs. We showed, using a range of supervised classification problems, that surrogate gradient learning in SNNs is robust to different shapes of surrogate derivatives. In contrast, inappropriate choice of scale adversely affected learning performance. Our results imply that for practical applications, surrogate derivatives should be appropriately normalized. Second, by constraining their activity through regularization, we showed that surrogate gradients could produce SNNs capable of efficient information processing with sparse spiking activity.

Surrogate gradients have been used by a number of studies to train SNNs (Neftci et al., 2019), solve small-scale toy problems with fractionally predictive neurons (Bohte, 2011), train convolutional SNNs on challenging neuromorphic (Amir et al., 2017; Orchard, Jayawant, Cohen, & Thakor, 2015) and vision benchmarks (Esser et al., 2016), or train recurrent SNNs on temporal problems requiring working memory (Bellec et al., 2018; Shrestha & Orchard, 2018). These studies used different surrogate derivatives ranging from exponential (Shrestha & Orchard, 2018), piece-wise linear (Bellec et al., 2018), or tanh (Woźniak et al., 2020), sometimes with a nonstandard neuron model with a constant leak term (Esser et al., 2016), but due to the different function choices and data sets, they are not easily comparable. Here we provide such a comprehensive comparison.

Towards this end, we had to make some compromises. Our study, like previous ones, is limited to supervised classification problems, because supervised learning offers a well-defined and intuitive quantification of computational performance. To keep the number of model parameters tractable, we focused on current-based LIF neurons. Moreover, we entirely dispensed with Dale’s law and relied solely on all-to-all connectivity. However, we believe that most of our findings will carry over to more realistic neuronal, synaptic, and connectivity models. Our study thus provides a set of blueprints and benchmarks to accelerate the design of future studies.

An alternative way of training SNNs, which does not rely on surrogate gradients, was introduced by Huh and Sejnowski (2018), who put forward a differentiable formulation of the neuronal spiking dynamics, thus allowing gradient descent with exact gradients. However, thus far, the approach has only been demonstrated for theta neurons, and nonleaky integrate-and-fire neurons and an extension to neuron models with nondifferentiable reset dynamics, like the LIF neurons we used here, is still pending.

Although considered biologically implausible (Crick, 1989), we have limited our study to training SNNs with backpropagation, the de facto standard for computing gradients in systems involving recurrence and hidden neurons. Although there exist more plausible forward-in-time algorithms like real-time recurrent learning (RTRL) (Williams & Zipser, 1989) they are prohibitively expensive or require additional approximations that affect learning performance (Bellec et al., 2019; Murray, 2019; Neftci et al., 2019; Zenke & Ganguli, 2018). Instead, using BPTT enabled the targeted manipulation of gradient flow through different elements of the network, which allowed us to dis-entwine some of the complexity of surrogate gradient learning. Finally, our study was purely numerical, and several important questions remain open. For instance, how does one optimally initialize hidden-layer weights? How well do these findings translate to convolutional neural networks? And why do the surrogate gradients work so well, despite ignoring the spike reset in their evaluation? Answering these questions requires additional numerical experiments and a rigorous theoretical understanding of surrogate gradient learning in SNNs, both of which remain for future work.

In summary, surrogate gradients allow translating the success of deep learning to biologically inspired SNNs by optimizing their connectivity toward functional complexity through end-to-end optimization. The in-depth study of both surrogate gradients and the resulting functional SNNs will occupy scientists for years to come and will likely prove transformative for neural circuit modeling.

## 4 Methods

### 4.1 Supervised Learning Tasks

In this study, we used a number of synthetic and real-world learning tasks with the overarching aim of balancing computational feasibility and practical relevance. To that end, we focused on synthetic data sets generated from random manifolds and real-world auditory datasets.

#### 4.1.1 Smooth Random Manifold Data Sets

We generated a range of synthetic classification data sets based on smooth random manifolds. Suppose we want to generate a smooth random manifold of dimension *D* in an embedding space of dimension *M*. We are looking for a smooth random function *f* : ℝ^*D*^ → ℝ^*M*^ defined over the finite interval 0 ≤ *x* < 1 in each intrinsic manifold coordinate axis. Moreover, we would like to keep its values bounded in a similar (0 ≤ *x < τ*_randman_)^*M*^ box in the embedding space. To achieve this, we first generate *M* smooth random functions *f*_*i*_ : ℝ^*D*^ → ℝ and then combine them to *f* : ℝ^*D*^ → ℝ^*M*^. Specifically, we generate the *f*_*i*_ from the Fourier basis as follows:

$$f_{i}(\vec{x})=\underset{j\in D}{\prod }\left[\sum_{k=1}^{n_{\text{cutoff}\;}}\frac{1}{k^{\alpha }}\theta_{ijk}^{\text{A}}\sin\left(2\pi \left(kx_{j}\theta_{ijk}^{\text{B}}+\theta_{ijk}^{\text{C}}\right)\right)\right],$$

where the parameters $\theta_{ijk}^{L}$ for *L* ∈ {A, B, C} were drawn independent and identically distributed from a uniform distribution 𝒰 (0, 1). We set *n*_cutoff_ = 1000, which leaves *α* as a parameter that controls the smoothness of the manifold. Larger values of *α* lead to more slowly varying manifolds, whereas smaller values increase the high-frequency content (see Figure 1a). In addition to *α*, the complexity of the learning problem can be seamlessly adjusted by either increasing the intrinsic dimension *D* of the manifold or the number of random manifolds whereby each random manifold corresponds to a separate class of the classification problem (see Figure 1b).

Concretely, we generated spike trains by randomly and uniformly sampling points from a *D*-dimensional hypercube with a side length of one. We then interpreted these points as intrinsic manifold coordinates and converted them to the corresponding embedding space coordinates. We next standardized these values along all embedding dimensions to lie between 0 and *τ*_randman_ and interpreted these *M*-dimensional coordinates as the firing times of *M* distinct input neurons belonging to the same class of the classification problem (see Figure 1c). Example code to generate smooth random manifolds following this procedure is available at https://github.com/fzenke/randman.

We chose a default parameter set for most of our experiments that struck a good balance between minimizing both the embedding dimension and the number of samples needed to solve the problem while simultaneously not being solvable by a two-layer network without hidden units. Specifically, we chose a 10-way problem with *D* = *α* = 1, *M* = 20, and *τ*_randman_ = 50 ms and fixed the random seed in situations in which we reused the same data set. In practice, the spike time $t_{i}^{c}$ of input neuron *i* in a given trial of class *c* is $t_{i}^{c}=f_{i}^{c}(X)$, where the $f_{i}^{c}$ are fixed random functions and *X* is a uniformly distributed random number between zero and one. For all simulation experiments, we generated 1000 data points for each class, out of which we used 800 for training and two sets of 100 each for validation and testing purposes.

#### 4.1.2 Spike Latency MNIST Data Set

To convert the analog valued MNIST data set (LeCun, Cortes, & Burges, 1998) to firing times, we proceeded as follows. We first standardized all pixel values $x_{i}^{\mu }$ to lie within the interval 0 ≤ *x* < *τ*_eff_. We then computed the time to first spike latency *T* as the time to reach firing threshold of a leaky integrator,

$$T(x)=\{\begin{array}{ll} \tau_{\text{eff}}\log\left(\frac{x}{x-\vartheta }\right) & x>\vartheta \\ \infty & \text{otherwise} \end{array}$$

and in our simulations, we used *ϑ* = 0.2 and *τ*_eff_ = 50 ms (see Figures 7a and 7b).

#### 4.1.3 Auditory Data Sets

We used both spiking and nonspiking auditory data sets of digit and word utterances. Specifically, we used the SHDs without any further preprocessing (Cramer, Stradmann et al., 2020). For performance reasons and to dispense with the spike conversion process, we ran additional simulations with nonspiking auditory inputs. Specifically, we worked with the raw Heidelberg Digits (RawHD) and Pete Warden’s Speech Commands data set (RawSC) (Warden, 2018) which were preprocessed as follows. We first applied a preemphasis filter to the raw audio signal *x*(*t*) by computing *y*(*t*) = *x*(*t*) − 0.95*x*(*t* −1). We then computed 25 ms frames with a 10 ms stride from the resulting signal and applied a Hamming window to each frame. For each frame, we computed the 512-point fast Fourier transform to obtain its power spectrum. From the power spectrum, we further computed the filter banks by applying 40 triangular filters on a Mel-scale (Huang, Acero, Hon, & Reddy, 2001). After cropping or padding to 80 (RawHD) or 100 (RawSC) steps by repeating the last frame, the analog-valued filter banks were fed directly to the SNNs.

### 4.2 Network Models

To train SNN models with surrogate gradients, we implemented them in PyTorch (Paszke et al., 2019). To that end, all models were explicitly formulated in discrete time with time step Δ*t*.

#### 4.2.1 Neuron Model

We used leaky integrate-and-fire neurons with current-based exponential synapses (Gerstner, Kistler, Naud, & Paninski, 2014; Vogels & Abbott, 2005). The membrane dynamics of neuron *i* in layer *l* were characterized by the following update equations,

(4.1) $${𝒰}_{i}^{\left(l\right)}[n+1]=\left(\beta_{\text{mem}\;}{𝒰}_{i}^{\left(l\right)}[n]+\left(1-\beta_{\text{mem}\;}\right)I_{i}^{\left(l\right)}[n]\right)\left(1-S_{i}^{\left(l\right)}[n]\right),$$

where ${𝒰}_{i}^{\left(l\right)}[n]$ corresponds to the membrane potential of neuron *i* in layer *l* at time step *n* and is its $S_{i}^{\left(l\right)}[n]$, the associated output spike train defined via the Heaviside step function Θ as $S_{i}^{\left(l\right)}[n]\equiv \text{Θ}\left({𝒰}_{i}^{\left(l\right)}[n]-1\right)$. Note that in this formulation, the membrane dynamics are effectively rescaled such that the resting potential corresponds to zero and the firing threshold of one. This choice simplifies the implementation of the neuronal reset dynamics through the factor on the right-hand side. During the backward pass of gradient computation with BPTT, the derivative of the step function is approximated using a surrogate, as explained later. In situations in which we ignored the reset term, this was done differently for the output spike train and the spike train underlying the reset term by detaching it from the computational graph. The membrane decay variable *β*_mem_ is associated with the membrane time constant *τ*_mem_ through $\beta_{\text{mem}}\equiv \exp\left(-\frac{\text{Δ}t}{\tau_{\text{mem}}}\right)$. Finally, the variable $I_{i}^{\left(l\right)}$ is the synaptic current defined as

$$I_{i}^{\left(l\right)}[n+1]=\beta_{\text{syn}}I_{i}^{\left(l\right)}[n]+\underset{j}{\sum }W_{ij}^{\left(l\right)}S_{j}^{\left(l-1\right)}[n]+\underset{j}{\sum }V_{ij}^{\left(l\right)}S_{j}^{\left(l\right)}[n],$$

with feedforward afferent weights *W*_*ij*_ and the optional recurrent weights *V*_*ij*_. In analogy to the membrane decay constant, *β*_syn_ is defined as $\beta_{\text{syn}}\equiv \exp\left(-\frac{\text{Δ}t}{\tau_{\text{syn}\;}}\right)$ We set *τ*_mem_ = 10 ms and *τ*_syn_ = 5 ms. Together the computations involved in each time step can be summarized in the computational graph of the model (see Figure 4).

#### 4.2.2 Readout Layer

The readout units in our models are identical to the above neuron model, but without the spike and associated reset. Additionally, we allow for a separate membrane time constant *τ*_readout_ = 20 ms with $\beta_{\text{out}}\equiv \exp\left(-\frac{\text{Δ}t}{\tau_{\text{readout}\;}}\right)$. Overall, their dynamics are described by

$$U_{i}^{\text{(out)}}[n+1]=\beta_{\text{out}}U_{i}^{\text{(out)}}[n]+\left(1-\beta_{\text{out}}\right)I_{i}^{\text{(out)}}[n].$$

#### 4.2.3 Connectivity and Initialization

We used all-to-all connectivity in all simulations without bias terms unless mentioned explicitly. The weights were initialized from a uniform distribution $U(-\sqrt{k},\sqrt{k})\;$ with $k=\frac{1}{n_{\text{inputs}\;}}$ where *n*_inputs_ is the number of afferent connections.

#### 4.2.4 Readout Heads and Supervised Loss Function

We trained all our networks by minimizing a standard cross-entropy loss *ℒ*_sup_ defined as

$${ℒ}_{\sup}=-\frac{1}{N}\sum_{\mu =1}^{N}\sum_{i=1}^{C}y_{i}^{\mu }\log\left(p_{i}^{\mu }\right),$$

where $y_{i}^{\mu }$ is the one-hot encoded target for input *μ, N* is the number of input samples, and *C* is the number of classes. The output probabilities $p_{i}^{\mu }$ were given by the Softmax function

$$p_{i}^{\mu }=\frac{e^{a_{i}^{\mu }}}{\sum_{k=1}^{C}e^{a_{k}^{\mu }}},$$

in which the logits $a_{i}^{\mu }$ for each input *μ* were depending on the readout configuration either given by the sum over all time steps $a_{i}^{\mu }=\sum_{n}\left({𝒰}_{i}^{\text{(out)}\;}[n]\right)$ or defined as the maximum $a_{i}^{\mu }=\max_{n}\left({𝒰}_{i}^{\text{(out)}\;}[n]\right)$, a notion inspired by the Tempotron (Gütig & Sompolinsky, 2006). It is worth noting that in the case of the Tempotron, the maximum over time is combined with a hinge loss function, which allows for binary classification only. In contrast, the Softmax formulation that we used throughout this letter enables our framework to perform multiway classification.

#### 4.2.5 Activity Regularization

To control spiking activity levels in the hidden layers, we employed two forms of activity regularization. First, to prevent quiescent units in the hidden layers, we introduced a lower-activity threshold *ν*_lower_ at the neuronal level defined as

$$g_{\text{lower}\;}^{\mu }=\frac{\lambda_{\text{lower}\;}}{M}\sum_{i}^{M}{\left({\left[v_{\text{lower}\;}-\zeta_{i}^{(l),\mu }\right]}_{+}\right)}^{2},$$

with the neuronal spike count $\zeta_{i}^{\left(l\right)}\equiv \left(\sum_{n}S_{i}^{\left(l\right)}[n]\right)$ and the number of neurons *M* in hidden-layer *l*. Similarly, we defined an upper threshold at the population level as

$$g_{\text{upper}\;}^{\mu }=-\lambda_{\text{upper}\;}{\left({\left[\frac{1}{M}\sum_{i}^{M}\zeta_{i}^{(l),\mu }-v_{\text{upper}\;}\right]}_{+}\right)}^{L},$$

for which we explored both values of *L* ∈ {1, 2}. The overall regularization loss was computed by summing and averaging ${ℒ}_{\text{reg}\;}=\frac{1}{N}\sum_{\mu }\left(g_{\text{lower}\;}^{\mu }+g_{\text{upper}\;}^{\mu }\right)$. Finally we optimized the total loss *ℒ*_tot_ = *ℒ*_sup_ + *ℒ*_reg_ by dint of surrogate gradient descent.

#### 4.2.6 Surrogate Gradient Descent

We minimized the loss *ℒ*_tot_ by adjusting the parameters *W* and *V* in the direction of the negative surrogate gradients using Adam with default parameters (Kingma & Ba, 2014). Surrogate gradients were computed with backpropagation through time using PyTorch’s automatic differentiation capabilities. To deal with the nondifferential spiking nonlinearity of the hidden-layer neurons, we approximated their derivatives $S^{\prime }\left({𝒰}_{i}^{\mu }[n]\right)={\text{Θ}}^{\prime }\left({𝒰}_{i}^{\mu }[n]-1\right)$ with suitable surrogates $\sigma^{\prime }\left({𝒰}_{i}^{\mu }[n]\right)=h\left({𝒰}_{i}^{\mu }[n]-1\right)$. Throughout, we used the following functions *h*(*x*):

**SuperSpike:** $h(x)=\frac{1}{{\left(\beta |x|+1\right)}^{2}}$

**Sigmoid′:** *h*(*x*) = *s*(*x*)(1 − *s*(*x*)) with the sigmoid function $s(x)=\frac{1}{1+\exp(-\beta x)}$

**Esser et al.:** *h*(*x*) = max(0, 1,0 − *β*|*x*|)

where *β* is a parameter that controls the slope of the surrogate derivative. In Figure 5, we additionally considered an asymptotic variant of SuperSpike defined as $h(x)=\frac{\beta }{{\left(\beta |x|+1\right)}^{2}}$. Unless mentioned otherwise, we set *β* = 10. Table 1 specifies the relevant hyperparameters used in the different simulations. An example of how these manipulations can be achieved easily with PyTorch can be found at https://github.com/fzenke/spytorch (Zenke, 2019). All simulations and parameter sweeps were performed on compute nodes equipped with Nvidia Quadro RTX 5000 and V100 GPUs.

## Figures and Tables

**Table 1:** Parameter Values of Network Simulations.

Parameter	Randman	MNIST	SHD	RawHD	RawSC
Number of input units	20	784	700	40	40
Number of hidden units	100	100	256	256	256
Number of readout units	10	10	20	20	35
Δ*t* / Number of steps	1ms / 100	1ms / 100	2ms / 500	2ms / 80	2ms / 100
Minibatch size	128,250	256	256	128	512
Number of epochs	50–100	100	200	50	50
Data set (train/valid./test)	8k/2k/2k	45k/5k/10k	7498/833/2088	7498/833/2088	84849/9981/11005
Number of hidden layers *n*_h_	≤ 2	≤ 3	≤ 2	1	1
Learning rate sweep	10^−3^ ≤ *η* ≤ 0.1	2 × 10^−4^ ≤ *η* ≤1^−1^	2 × 10^−4^ ≤ *η* ≤1^−1^	1 × 10^−3^	1 × 10^−3^, 5 × 10^−3^, 0.01
Best *η*	0.05	0.01	1 × 10^−3^	1 × 10^−3^	1 × 10^−3^
Lower L2: λ_lower_/*ν*_lower_	100.0/10^−3^	—	100.0/10^−3^	100.0/10^−3^	100.0/(0.01, 10^−3^)
Upper L1 strength λ_upper,1_	1,100	0–100	0.06	0, 10, 20, 100	0.06, 1, 10
Upper L1 threshold *ν*_upper,1_	0–1000	0–1000	0–1000	0, 0.1, 0.2, 0.5, 1, 100	0–1000
Upper L2 strength λ_upper,2_	0,1,100	0, 0.001, 0.1, 1	—	—	0,1
Upper L2 threshold *ν*_upper,1_	0–100	0.1, 100	—	—	10,100

## References

[R1] Amir A, Taba B, Berg D, Melano T, McKinstry J, Di Nolfo C, Kusnitz J (2017). A low power, fully event-based gesture recognition system.

[R2] Barrett DG, Morcos AS, Macke JH (2019). Analyzing biological and artificial neural networks: Challenges with opportunities for synergy?. Current Opinion in Neurobiology.

[R3] Bellec G, Salaj D, Subramoney A, Legenstein R, Maass W, Bengio S, Wallach H, Larochelle H, Grauman K, Cesa-Bianchi N, Garnett R (2018). Advances in neural information processing systems.

[R4] Bellec G, Scherr F, Hajek E, Salaj D, Legenstein R, Maass W (2019). Biologically inspired alternatives to backpropagation through time for learning in recurrent neural nets. arXiv.

[R5] Boahen K (2017). Aneuromorph’s prospectus. Comput Sci Eng.

[R6] Bohte SM (2011). Artificial Neural Networks and Machine Learning—ICANN 2011.

[R7] Cramer B, Billaudelle S, Kanya S, Leibfried A, Grübl A, Karasenko V, Zenke F (2020). Training spiking multi-layer networks with surrogate gradients on an analog neuromorphic substrate. arXiv.

[R8] Cramer B, Stradmann Y, Schemmel J, Zenke F (2020). The Heidelberg spiking data sets for the systematic evaluation of spiking neural networks. IEEE Transactions on Neural Networks and Learning Systems.

[R9] Crick F (1989). The recent excitement about neural networks. Nature.

[R10] Cueva CJ, Marcos E, Saez A, Genovesio A, Jazayeri M, Romo R, Fusi S (2019). Low dimensional dynamics for working memory and time encoding. bioRxiv.

[R11] Esser SK, Merolla PA, Arthur JV, Cassidy AS, Appuswamy R, Andreopoulos A, Modha DS (2016). Convolutional networks for fast, energy-efficient neuromorphic computing. Proc Natl Acad Sci USA.

[R12] Gerstner W, Kistler WM, Naud R, Paninski L (2014). Neuronal dynamics: From single neurons to networks and models of cognition.

[R13] Gütig R (2016). Spiking neurons can discover predictive features by aggregate-label learning. Science.

[R14] Gütig R, Sompolinsky H (2006). The tempotron: A neuron that learns spike timing-based decisions. Nat Neurosci.

[R15] He K, Zhang X, Ren S, Sun J (2015). Delving deep into rectifiers: Surpassing human-level performance on imagenet classification.

[R16] Hochreiter S (1998). The vanishing gradient problem during learning recurrent neural nets and problem solutions. Int J Unc Fuzz Knowl Based Syst.

[R17] Huang X, Acero A, Hon H-W, Reddy R (2001). Spoken language processing: A guide to theory, algorithm and system development.

[R18] Huh D, Sejnowski TJ, Bengio S, Wallach H, Larochelle H, Grauman K, Cesa-Bianchi N, Garnett R (2018). Advances in neural information processing systems.

[R19] Hunsberger E, Eliasmith C (2015). Spiking deep networks with LIF neurons. arXiv.

[R20] Kingma D, Ba J (2014). Adam: A method for stochastic optimization. arXiv.

[R21] LeCun Y, Bengio Y, Hinton G (2015). Deep learning. Nature.

[R22] LeCun Y, Cortes C, Burges CJ (1998). The MNIST database of handwritten digits.

[R23] Lee JH, Delbruck T, Pfeiffer M (2016). Training deep spiking neural networks using backpropagation. Front Neurosci.

[R24] Maheswaranathan N, McIntosh LT, Kastner DB, Melander J, Brezovec L, Nayebi A, Baccus SA (2018). Deep learning models reveal internal structure and diverse computations in the retina under natural scenes. bioRxiv.

[R25] Mante V, Sussillo D, Shenoy KV, Newsome WT (2013). Context-dependent computation by recurrent dynamics in prefrontal cortex. Nature.

[R26] McClure P, Kriegeskorte N (2016). Representational distance learning for deep neural networks. Front Comput Neurosci.

[R27] McIntosh L, Maheswaranathan N, Nayebi A, Ganguli S, Baccus S, Lee D, Sugiyama M, Luxburg U, Guyon I, Garnett R (2016). Advances in neural information processing systems.

[R28] Michaels JA, Schaffelhofer S, Agudelo-Toro A, Scherberger H (2019). A neural network model of flexible grasp movement generation. bioRxiv.

[R29] Mishkin D, Matas J (2016). All you need is a good init. arXiv.

[R30] Mostafa H (2018). Supervised learning based on temporal coding in spiking neural networks. Trans Neural Netw Learn Syst.

[R31] Murray JM (2019). Local online learning in recurrent networks with random feedback. eLife.

[R32] Neftci EO (2018). Data and power efficient intelligence with neuromorphic learning machines. iScience.

[R33] Neftci EO, Mostafa H, Zenke F (2019). Surrogate gradient learning in spiking neural networks: Bringing the power of gradient-based optimization to spiking neural networks. IEEE Signal Process Mag.

[R34] Orchard G, Jayawant A, Cohen GK, Thakor N (2015). Converting static image datasets to spiking neuromorphic datasets using saccades. FrontNeurosci.

[R35] Paszke A, Gross S, Massa F, Lerer A, Bradbury J, Chintala S, Wallach H, Larochelle H, Beygelzimer A, d’Alché-Buc F, Fox E, Garnett R (2019). Advances in neural information processing systems.

[R36] Pfeiffer M, Pfeil T (2018). Deep learning with spiking neurons: Opportunities and challenges. Front Neurosci.

[R37] Pospisil DA, Pasupathy A, Bair W (2018). “Artiphysiology” reveals V4-like shape tuning in a deep network trained for image classification. eLife.

[R38] Richards BA, Lillicrap TP, Beaudoin P, Bengio Y, Bogacz R, Christensen A, Kording KP (2019). A deep learning framework for neuroscience. Nat Neurosci.

[R39] Roy K, Jaiswal A, Panda P (2019). Towards spike-based machine intelligence with neuromorphic computing. Nature.

[R40] Schemmel J, Briiderle D, Griibl A, Hock M, Meier K, Millner S (2010). A wafer-scale neuromorphic hardware system for large-scale neural modeling.

[R41] Schmidhuber J (2015). Deep learning in neural networks: An overview. Neural Netw.

[R42] Shrestha SB, Orchard G, Bengio S, Wallach H, Larochelle H, Grauman K, Cesa-Bianchi N, Garnett R (2018). Advances in neural information processing systems.

[R43] Sterling P, Laughlin S (2017). Principles of neural design.

[R44] Stroud JP, Porter MA, Hennequin G, Vogels TP (2018). Motor primitives in space and time via targeted gain modulation in cortical networks. Nature Neuroscience.

[R45] Sussillo D, Barak O (2012). Opening the black box: Low-dimensional dynamics in high-dimensional recurrent neural networks. Neural Comput.

[R46] Tanaka H, Nayebi A, Maheswaranathan N, McIntosh L, Baccus S, Ganguli S, Wallach H, Larochelle H, Beygelzimer A, d’Alché-Buc F, Fox E, Garnett R (2019). Advances in neural information processing systems.

[R47] Vogels TP, Abbott LF (2005). Signal propagation and logic gating in networks of integrate-and-fire neurons. J Neurosci.

[R48] Wang J, Narain D, Hosseini EA, Jazayeri M (2018). Flexible timing by temporal scaling of cortical responses. Nat Neurosci.

[R49] Warden P (2018). Speech commands: A dataset for limited-vocabulary speech recognition. arXiv.

[R50] Williams RJ, Zipser D (1989). A learning algorithm for continually running fully recurrent neural networks. Neural Computation.

[R51] Williamson RC, Doiron B, Smith MA, Yu BM (2019). Bridging large-scale neuronal recordings and large-scale network models using dimensionality reduction. Current Opinion in Neurobiology.

[R52] Woźniak S, Pantazi A, Bohnstingl T, Eleftheriou E (2020). Deep learning incorporating biologically inspired neural dynamics and in-memory computing. Nature Machine Intelligence.

[R53] Yamins DLK, DiCarlo JJ (2016). Using goal-driven deep learning models to understand sensory cortex. Nat Neurosci.

[R54] Yamins DLK, Hong H, Cadieu CF, Solomon EA, Seibert D, DiCarlo JJ (2014). Performance-optimized hierarchical models predict neural responses in higher visual cortex. Proc Natl Acad Sci USA.

[R55] Zenke F (2019). SpyTorch.

[R56] Zenke F, Ganguli S (2018). SuperSpike: Supervised learning in multilayer spiking neural networks. Neural Comput.

[R57] Zimmer R, Pellegrini T, Singh SF, Masquelier T (2019). Technical report: Supervised training of convolutional spiking neural networks with PyTorch.

